# Ossification of the Dental Pulp

**Published:** 1853-04

**Authors:** 


*Ossification of the Dental Pulp.*—We have recently received from Dr. Hardtner, of Illinois, a molar tooth, furnishing a beautiful example of ossification of the dental pulp. We have several other specimens of the same kind, which we intend to place in the Museum of the Baltimore College of Dental Surgery.

